# Everybody's Page

**Published:** 1890-02-08

**Authors:** 


### EVERYBODY'S PAGE.

Drunkenness is said to be on the increase in Russia.

In Vienna the incandescent lamp has been much used in medical experiments on horses for diseases of the nostril. There is an excellent cooling arrangement which allows cold water to circulate round the lamp.

Coca is largely grown in the Andes. The largest depot is La Paz, in Bolivia. When cocaine was first discovered the leaves were sent straight to Europe, but the sea voyage was found to rob the plant of the alkaloids, so these are now extracted in South America and then exported for use.

Dairy management has attracted much attention lately in Denmark, where State Butter Shows are now being organized on an extensive scale. The Consul at Copenhagen reports that a continuous butter show is to be held at the expense of the State during several months in each year; fresh samples of butter are to be received every 14 days.

We are sometimes inclined to think that some of the human race are like pussy, possessed of nine lives, but the people Dr. Norman Kerr has come across must possess a still more marvellous supply. He has known men and women to so habituate themselves to eating arsenic that they can take 10 to 15 grains daily and say they feel all the better for it. Ladies think they improve their complexion by eating it, and men use it as a general tonic.

Dr. Hanau, of Zurich, has successfully propagated cancer in rats by inoculation.

Out of 366 suicides in Vienna in 1889, ninety-two were women.

If this catches the eye of any budding youth he will be grateful that Providence has made England his home, and not South Carolina, for that State has passed a law which forbids the sale of cigarettes to minors, under pain of a severe penalty if the law be infringed. We are inclined to think it rather a good law!

The *Boston Post* relates a curious story of the propagation of scarlatina. A picture book which had been used by a little boy in America, who died of scarlatina, was packed away in a trunk for twenty-six years. After a lapse of this time the book was sent over to England and given to a boy of two years of age, who a fortnight after receiving it sickened of scarlatina.

Few people probably were aware of the existence of enormous deposits of borax in California, till Mr. C. Napier Hake read his paper before the Society of Chemical Industry. Some 450 miles from San Francisco there is a dry salt lake about 12 miles long and eight miles broad, in the valley of the Slate Range and Argus Mountains. This lake yields bi-carbonate of soda, common salt, and borax. Beneath the incrustation is a deep bed of black mud. Formerly the supply of borax was obtained from Thibet and the north of India, until Italy and Peru became sources of supply.

